# Supplementary material for: Circadian Disruption Primes Myofibroblasts for Accelerated Activation as a Mechanism Underpinning Fibrotic Progression in Non-Alcoholic Fatty Liver Disease
Source: Cells. 2023 Jun 8;12(12):1582. doi: 10.3390/cells12121582 (PMC10297459; doi:10.3390/cells12121582)
Supplement: Supplementary file 1 [file cells-12-01582-s001.zip › Supplementary Figures for CLOCK paper.pdf]

Supplementary Figures for Circadian disruption primes myofibroblasts for accelerated activation as a mechanism underpinning fibrotic progression in non-alcoholic fatty liver disease

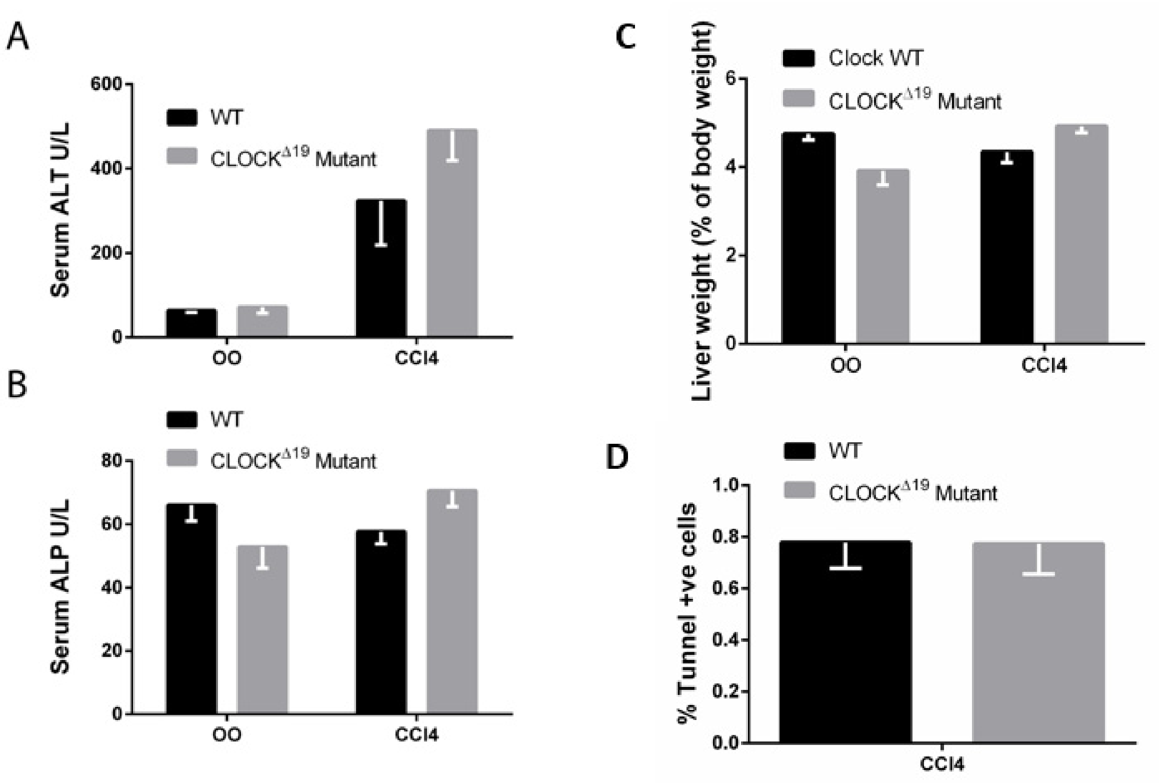

Supplementary Figure S1. Liver weights and function tests after CCl4 treatment No significant differences between the WT and CLOCKΔ19 are evident in (A) serum LFT, (B) Serum ALP, (C) liver weight to body weight ratios or (D) TUNEL staining. Error bars represent mean +/- SD, n=3-5.

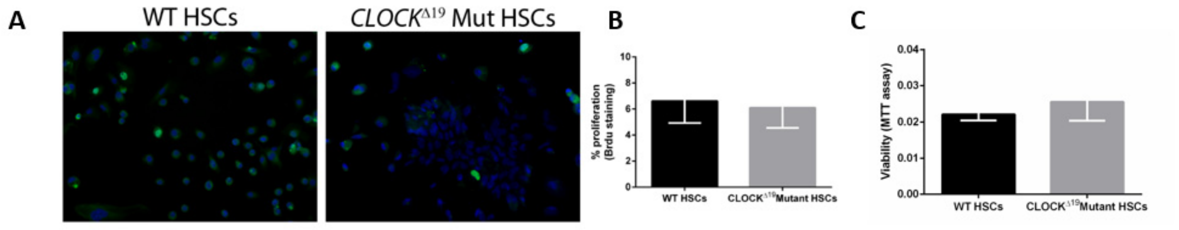

Supplementary Figure S2. BRDU incorporation and MTT assay No significant difference between WT and CLOCKΔ19 was observed in proliferation via BRDU (A,B) or viability via MTT assay. Error bars represent mean +/- SD. N=3
